# Response to Different Oxygen Partial Pressures and Evolution Analysis of Apoptosis-Related Genes in Plateau Zokor (*Myospalax baileyi*)

**DOI:** 10.3389/fgene.2022.865301

**Published:** 2022-06-08

**Authors:** Zhifang An, Xiaoqi Chen, Jimei Li

**Affiliations:** ^1^ State Key Laboratory of Plateau Ecology and Agriculture, Qinghai University, Xining, China; ^2^ Department of Obstetrics and Gynaecology, Affiliated Hospital of Qinghai University, Xining, China; ^3^ Research Center for High Altitude Medicine, Qinghai University, Xining, China; ^4^ Department of General Medicine, Qinghai Provincial People’s Hospital, Xining, China

**Keywords:** plateau zokor (*Myospalax baileyi*), apoptosis-related genes, evolution analysis, hypoxia, oxygen partial pressure

## Abstract

The plateau zokor (*Myospalax baileyi*) is a native species of the Qinghai–Tibet Plateau that spends its entire life underground in sealed burrows with hypoxic conditions. The present study aimed to assess the sequence characteristics of apoptosis-related genes and the response to different oxygen partial pressures (pO_2_) in plateau zokor and Sprague-Dawley rats. The sequences of the p53-induced protein with a death domain (Pidd), p53-upregulated modulator of apoptosis (Puma), insulin-like growth factor binding protein 3 (Igfbp3), and apoptosis protease-activating factor 1 (Apaf1) were evaluated concerning homology and convergent evolution sites, and their mRNA levels were evaluated in different tissues under 14.13 (3,300 m) and 16.12 kPa (2,260 m) pO_2_ conditions. Our results showed that, (1) the sequences of the apoptosis-related genes in plateau zokor were highly similar to those of *Nannospalax galili*, followed by *Rattus norvegicus*; (2). Pidd, Puma, Igfbp3, and Apaf1 of plateau zokor were found to have five, one, two, and five convergent sites in functional domains with *N. galili*, respectively. Lastly (3), under low pO_2_, the expression of *Pidd* and *Puma* was downregulated in the lung of plateau zokors. In turn, *Igfbp3* and *Apaf1* were upregulated in the liver and lung, and *Puma* was upregulated in the skeletal muscle of plateau zokor under low pO_2_. In Sprague-Dawley rats, low pO_2_ downregulated *Puma* and *Apaf1* expression in the liver and downregulated *Igfbp3* and *Puma* in the lung and skeletal muscle separately. In contrast, low pO_2_ upregulated *Pidd* expression in the liver and skeletal muscle of Sprague-Dawley rats. Overall, the expression patterns of *Apaf1*, *Igfbp3*, and *Puma* showed the opposite pattern in the liver, lung, and skeletal muscle, respectively, of plateau zokor as compared with Sprague-Dawley rats. In conclusion, for the long-time adaptation to hypoxic environments, *Pidd*, *Puma*, *Igfbp3*, and *Apaf1* of plateau zokor underwent convergent evolution, which we believe may have led to upregulation of their levels under low oxygen partial pressures to induce apoptosis, so as to suppress tumorigenesis under hypoxic environments in plateau zokor.

## Introduction

Under hypoxic conditions, cells commonly initiate the process of programmed death via apoptosis, in which two pathways are involved: the extrinsic pathway (death receptor pathway) and intrinsic pathway (mitochondrial pathway) (Pan et al., 2014; Lohberger et al., 2016). In the extrinsic pathway, the death-inducing signaling complex (DISC) is stimulated, which will in turn promote the hydrolysis of caspase that has a critical function, to transmit the apoptotic signal into the nucleus land trigger apoptosis. In the intrinsic pathway, hypoxia induces apoptosis via the increased permeability of the mitochondrial membrane, specifically by directly inhibiting the electron transport chain of the mitochondrial inner membrane, thereby causing cytochrome c released to the cytoplasm (Pan et al., 2014; Sendoel and Hengartner, 2014; Lohberger et al., 2016).

Proapoptotic and antiapoptotic genes are also key regulators of the apoptotic process, including the proapoptotic genes B-cell lymphoma 2 (Bcl-2)-associated X protein (Bax), Bcl-xl/Bcl-2-associated death promoter, Bcl-2 homology 3 (BH3)-only proapoptotic protein (Noxa), Bcl2/adenovirus EIB 19kD-interacting protein 3 (Bnip3), p53-induced protein with a death domain (Pidd), p53-upregulated modulator of apoptosis (Puma), insulin-like growth factor binging protein 3 (Igfbp3), apoptotic protease-activating factor-1 (Apaf1), and the antiapoptotic Bcl-2 and Bcl-2-like 14 protein (Hockenbery et al., 1993; Gross et al., 1999; Mcclintock et al., 2002; Gogvadze and Orrenius, 2006). Studies have shown that hypoxia treatment promotes the upregulation of Bax and downregulation of Bcl-2 in myocardial and germ cells in rats (Nakamura et al., 2000; Liao et al., 2007). In addition, hypoxia can upregulate the levels of death receptor 5 (DR5), tumor necrosis factor-related apoptosis-inducing ligand (TRAIL), Fas, p53, and Bax; and downregulate the levels of cellular FADD-like interleukin-1β-converting enzyme-inhibitory protein (c-FLIP), decoy receptor 2 (DcR2), and Bcl-2 in murine spermatocytes (Yin et al., 2018). Moreover, hypoxia post-conditioning was found to downregulate the levels of *Puma* in neonatal rat cardiomyocytes (Li et al., 2015). Noteworthy, hypoxia, which is a signature of a solid tumor microenvironment, was found to lowly downregulate *Pidd* in tumors, including in hepatocellular carcinoma (HCC), testicular germ cell carcinoma, and lung cancer, as compared with normal tissues and cell lines (Kerley-Hamilton et al., 2005; Oliver et al., 2010; Shi et al., 2016).

Plateau zokor (*Myospalax baileyi*) is a wild subterranean rodent that lives in sealed burrows at an altitude of 2,800–4,200 m on the Qinghai–Tibet Plateau (Wang et al., 1979; Fan and Shi, 1982); thus, it spends its entire life underground in hypoxic and hypercapnic conditions (Fan and Shi, 1982; Nevo, 1999; Nevo et al., 2001; Shams et al., 2005), with an oxygen content 20% lower than that in the atmosphere (Zeng et al., 1984). Studies have shown that subterranean rodents have strong adapting capability, which grants them phenotypic, physiological, and molecular features to cope with the harsh burrowing environments (Arieli and Ar, 1981; Arieli et al., 1986; Widmer et al., 1997; Wang et al., 2008; Kim et al., 2011; Fang et al., 2014; Shao et al., 2015). To the best of our knowledge, subterranean rodents exhibit unique longevity and cancer resistance; for example, *Nannospalax galili* and *Heterocephalus glaber* are subterranean rodents that can live at least 21 years (Edrey et al., 2012) and more than 30 years (Buffenstein and Jarvis, 2002; Buffenstein, 2008; Kim et al., 2011), respectively. Indeed, tumors have never been observed in multi-years of observation of wild or captive subterranean rodents (Buffenstein and Jarvis, 2002; Buffenstein, 2008; Gorbunova et al., 2012; Delaney et al., 2013; Manov et al., 2013).

Previous studies have suggested that molecular pathways associated with hypoxia tolerance share common antiapoptotic functions with those related to tumor adaptivity in *Spalax* (Ashur-Fabian et al., 2004; Avivi et al., 2007; Band et al., 2010). Furthermore, transcriptome analysis demonstrated the presence of numerous apoptosis-related suppressors (Malik et al., 2011; Malik et al., 2012). p53 is a tumor suppressor protein that activates several target genes related to the cell cycle and apoptosis to inhibit the tumor’s growth (Avivi et al., 2005). Our previous research demonstrated that p53 is sensitive to the oxygen concentration in the tissues of plateau zokor, and hypoxia upregulates the levels of p53, whereas the Sprague–Dawley (SD) rat did not (An et al., 2018). In addition, a substitution in position 174 of *Spalax* spp. p53 sequence indicated that these rodents adapt to hypoxic environments by escaping from apoptosis via loss of function of apoptotic proteins, such as Apaf1, Puma, Noxa, and Bax (Ashur-Fabian et al., 2004; Avivi et al., 2005; Avivi et al., 2007). Indeed, a substitution at position 104 of p53 was shown to activate genes related to apoptosis, including *Igfbp3*, *Apaf1*, *Bax*, *Puma*, and *Noxa* in Gansu zokor (*Myospalax cansus*), as well as transcription of *Igfbp3*, *Apaf1*, and *Bax* in plateau zokor (Zhao et al., 2013). In addition, some apoptosis-related genes were evaluated in subterranean rodents, revealing that the expression of *Bcl2* in the lungs was significantly increased in chalk than in the basalt mole rat of *Spalax galili*, whereas *Apaf1*, *Bax*, *Igfbp3*, and *Puma* levels were similar in various tissues of the 2 mole rat populations (Zhao et al., 2016). Compared with normoxic conditions, hypoxic stress (6% O_2_ for 5 h) showed no effect on *Bnip3* expression in *Spalax galili* muscle and heart, whereas it was a slight increase under moderate oxygen levels (10% O_2_ for 22 or 44 h) was reported (Band et al., 2009). However, in *Rattus norvegicus*, the expression of *Bnip3* is significantly increased in the muscle and heart under such hypoxic conditions (Band et al., 2009). Despite several studies having explore the impact of hypoxia in apoptosis-related genes in hypoxia-tolerant subterranean rodents, most studies were conducted on *Spalax* spp., whereas little information is available on plateau zokor. To date, the response to different oxygen partial pressures in tissues of plateau zokor and the sequence characteristics of the apoptosis-related genes are not definitely established. Therefore, the present study aimed to assess the expression and sequence of apoptosis-related genes in plateau zokor and Sprague–Dawley rat tissues under different oxygen partial pressures to further understand the ability of plateau zokor to cope with oxygen partial pressures.

## Materials and Methods

### Animals and Sample Collection

Plateau zokors were live-trapped in the Zongjiagou region in Huangyuan country, Qinghai Province, China, at an altitude of 3,300 m with the oxygen partial pressure (pO_2_) of 14.13 kPa. Sprague–Dawley rats were bought from Lanzhou, Gansu Province, China. All animals were divided into two groups (1): 14.13 kPa (3,300 m) group, plateau zokors were captured in the field (3,300 m, the field was the habitats of plateau zokor), Sprague–Dawley rats were raised in the Zongjiagou region in Huangyuan country for 8 days (2); 16.12 kPa (2,260 m) group, plateau zokors were captured from Zongjiagou region in Huangyuan country and raised for 8 days in Xining City, Qinghai Province, China, at an altitude of 2,260 m with the oxygen partial pressure of 16.12 kPa. Sprague–Dawley rats were raised in Xining for 8 days. Experiments were performed on adult animals. Animals were housed in individual cages with sawdust and hay. The sample size was eight for each group. All animals were anesthetized with sodium pentobarbital (5%) and sacrificed using cervical dislocation immediately before dissection. Liver, lung, and skeletal muscle tissues were removed and immediately frozen in liquid nitrogen. All procedures involved in the handling and care of animals were in accordance with the China Practice for the Care and Use of Laboratory Animals and were approved by the China Zoological Society (permit number: GB/T35892-2018).

### RNA Extraction and Quantification of Apoptosis-Related Gene mRNA Using qRT-PCR

Total RNA was isolated from the liver, lung, and skeletal muscle tissues using TRIzol reagent (Invitrogen Corp, 15596026, United States). The concentration and purity were checked using UV spectrophotometry (1.8 < A260/A280 < 2.0). RNA integrity was assessed using electrophoresis. A reverse transcription reaction was synthesized starting with 3.8 μg of total RNA and the First Strand cDNA Synthesis kit (TIANGEN, KR118-02, China).

The quantitative real-time RT-PCR was performed on a Bio-Rad Connect real-time PCR detection system (Bio-Rad Laboratories, Hercules, CA, United States) using the SYBR Premix Ex Taq™ II (Takara Bio, RR820A, Japan) protocol to quantify the expression level of apoptosis-related genes in plateau zokors and Sprague–Dawley rats. The qRT-PCR was performed at 95°C for 3 min, and then for 40 cycles at 95°C for 30 s and 60°C for 30 s. The *β-actin* was used as an internal control. The relative expression level of apoptosis-related genes mRNA was computed based on the internal control gene using the 2_−△△Ct_ method (Livak and Schmittgen, 2001). The primers for apoptosis-related genes and *β-actin* were designed as follows: *M. baileyi Pidd*-F: 5′- CTA CCG TGA ACT ACA GCG TAT C -3′, *M. baileyi Pidd*-R: 5′- ACC TCT TCA GCC ACA TCC T -3’; *M. baileyi Puma*-F: 5′- CAG GGT GGG TGG TAA -3′, *M. baileyi Puma*-R: 5′- CGG GCG ACT CTA GGT GTT -3’; *M. baileyi Igfbp3*-F: 5′- TGG TGT GTG GAC AAG TAT G -3′, *M. baileyi Igfbp3*-R: 5′- AGT TCA CTT CGT CCT TCC -3’; *M. baileyi Apaf1*-F: 5′- GGA GAC TGA GGA GGT TGA AGA -3′, *M. baileyi Apaf1*-R: 5′- GCG TAT GCG GCT GGT AAT -3’; Rat *Pidd*-F: 5′- CCG TGA GGT AGT TGT GAG AA-3′, Rat *Pidd*-R: 5′- GGT AAT AGG CAG GTG TTG GA -3’; Rat *Puma*-F: 5′- TGT GGA GGA GGA GGA GTG -3′, Rat *Puma*-R: 5′- CGA TGT TGC TCT TCT TGT CTC -3’; Rat *Igfbp3*-F: 5′- TGA GGA GGA CCA CAA TGC -3′, Rat *Igfbp3*-R: 5′- GCT TAG ACT CGG AGG AGA AG -3’; Rat *Apaf1*-F: 5′- GTC ATC ACA GCA CCA TCC AGT A-3′, Rat *Apaf1*-R: 5′- TCA AGA ACG AGG AGC CAT CAG -3’; *β-actin*-F: 5′-TCA CCA ACT GGG ACG ATA TG -3′, *β-actin*-R: 5′-GTT GGC CTT AGG GTT CAG AG -3’.

Statistical analyses were performed using SAS 8.2 software. The expression levels of apoptosis-related genes between different oxygen partial pressures were compared using Student’s t-test. A value of *p* < 0.05 was considered to be statistically significant.

### Sequences of Apoptosis-Related Genes

The coding DNA sequences of apoptosis-related genes of plateau zokor and plateau pika (*Ochotona curzniae*) were obtained from the next generation sequencing databases and Isoform Sequencing databases, we were sequenced on Illumina Hiseq 4,000 and PacBio RSII platform and performed by Novogene Bioinformatics Technology Co., Ltd, Beijing, China. The sequences of the other 20 mammalian species were obtained from the NCBI (National Center for Biotechnology Information) (https://www.ncbi.nlm.nih.gov/), *N. galili*, *R. norvegicus*, *M. musculus*, *H. glaber*, *M. ochrogaster*, *M. auratus*, *C. griseus*, *J. jaculus*, *F. damarensis, C. porcellus, C. lanigera, O. degus, I. tridecemlineatus, O. princeps, B. Taurus*, *O. aries*, *C. hircus*, *H. sapiens*, *P. troglodytes*.

### Sequence Analysis

Nucleotide and amino acid sequences of apoptosis-related genes were aligned by using ClustalW2 (http://www.ebi.ac.uk/Tools/msa/clustalw2) and MEGA 7.0 software (Kumar et al., 2016) and manually adjusted with DNAMAN 9.0.

### Selection Pressure Analysis

ClustalX 1.81 software was used to sequence alignments (Jeanmougin et al., 1998), and MEGA 7.0 software was performed for format conversion (Kumar et al., 2016). The selection pressure analysis on apoptosis-related genes was estimated by using the maximum-likelihood method in codeml from the PAML 4.8 package based on the mammalian species tree. The omega (ω) values (dN/dS = nonsynonymous/synonymous) were calculated in specific lineages, the *ω* values indicate changes in selective pressures, with *ω* < 1, *ω* > 1, and *ω* = 1 indicates negative, positive, and neutral selections, respectively (Hurst, 2002; Xu and Yang, 2013). The branch-site models were used to investigate positive selective pressures in specific lineages (Zhang et al., 2005), including 22 mammalian species. The significance of the likelihood ratio test (LRT) statistic was determined using a χ2 distribution, and the positively selected sites were identified using Bayes Empirical Bayes (BEB) analysis (Zhang et al., 2005; Yang, 2007).

### Convergent Evolution Analysis

To infer convergent sites among branches of plateau zokor and *N. galili*, ancestral amino acid sequences were reconstructed using the Ancestors program in the MEGA software (Tamura et al., 2011). Eleven mammalian species were used to infer the convergent sites. Ancestral inferences appeared reliable because the posterior probabilities for the entire protein exceeded 99% for all nodes. We attempted to identify convergent changes by comparing ancestral and extant apoptosis-related gene protein sequences. Next, we calculated the probability that the observed convergent sites exceeded the expectation due to random chance using Jones-Taylor-Thornton (JTT) along with the Poisson models (Zhang and Kumar, 1997).

### Protein Structure Homology Modeling

We investigated the relationships between the amino acid substitution sites and the structural modeling of the apoptosis-related genes. The protein structures were constructed using the SWISS-MODEL server to assemble amino acids selected with a homology modeling protocol (Schwede et al., 2003). Then we downloaded the model with the high identity of the sequences from the SWISS-MODEL server and converted the proteins files in PDB format to PQR format with the PDB2PQR server (http://nbcr-222.ucsd.edu/pdb2pqr_2.1.1/) (Dolinsky et al., 2004; Dolinsky et al., 2007). The surface electrostatic potential of the protein structures was calculated and presented using PyMOL, VMD, and APBS (Humphrey et al., 1996; DeLano, 2002; Unni et al., 2011).

## Results

### Expression of Apoptosis-Related Genes in Tissues of Plateau Zokor and Sprague-Dawley Rats

**FIGURE 1:**
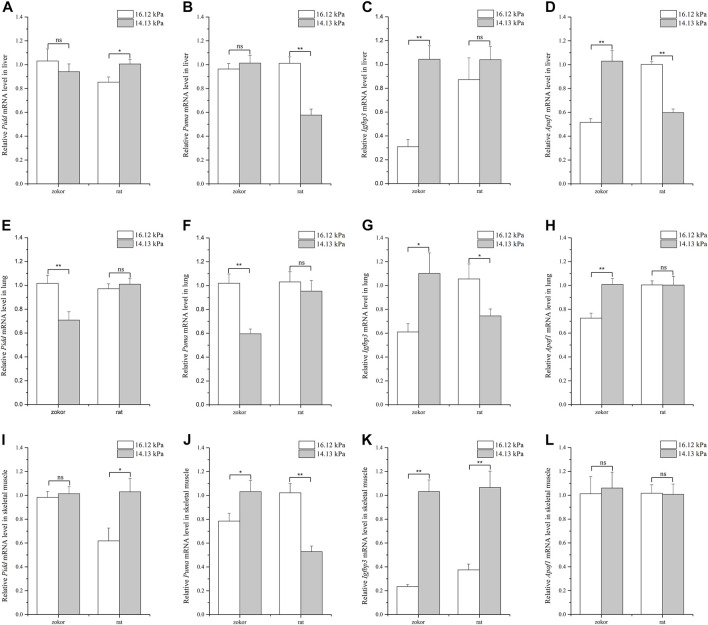
Quantification of apoptosis-related genes mRNA levels in tissues of plateau zokor and Sprague–Dawley rat under different oxygen partial pressures. Panels indicate liver **(A–D)**, lung **(E–H)**, and skeletal muscle **(I–L)**. ∗∗, *p* < 0.01; ∗, *p* < 0.05; ns, not significant (*p* > 0.05). The sample size was eight for each group.

Analysis of the levels of different apoptosis-related genes in the liver of plateau zokors and Sprague-Dawley rats showed that *Apaf1* expression pattern followed the expected trend so to cope with different pO_2_ conditions in plateau zokors: *Apaf1* was significantly higher under 14.13 kPa pO_2_ than at 16.12 kPa in plateau zokors, where it was significantly lower at 14.13 kPa pO_2_ in Sprague-Dawley rats (Figure 1D). Moreover, the relative expression of *Pidd* and *Puma* was not significantly different between 14.13 and 16.12 kPa, whereas that of *Igfbp3* was significantly higher at 14.13 kPa pO_2_ in plateau zokors (Figures 1A–C). In turn, in the liver of Sprague-Dawley rats, the expression of *Pidd* was significantly higher under 14.13 kPa pO_2_ than at 16.12 kPa (Figure 1A), whereas that of *Puma* was significantly lower at 14.13 kPa pO_2_ (Figure 1B).

In the lungs, the expression of *Pidd and Puma* was significantly lower under 14.13 kPa pO_2_ than at 16.12 kPa in plateau zokors, where that of *Igfbp3* and *Apaf1* was significantly higher at 14.13 kPa pO_2_ (Figures 1E–H). In contrast, the expression of *Igfbp3* was significantly lower under 14.13 kPa pO_2_ than 16.12 kPa in Sprague-Dawley rats (Figure 1G), whereas that of *Pidd*, *Puma*, and *Apaf1* remained unaltered regardless of the pO_2_ condition (Figures 1E,F,H).

Lastly, the levels of genes related to apoptosis were measured in samples of skeletal muscle. Overall, the relative expression of *Pidd* and *Apaf1* was found to be similar under different pO_2_ conditions whereas that of *Puma* and *Igfbp3* was significantly higher under 14.13 kPa pO_2_ than at 16.12 kPa in plateau zokors (Figure 1I **, 1L, 1J, 1K**). In Sprague-Dawley rats, the level of *Pidd* and *Igfbp3* was significantly higher at 14.13 kPa pO_2_ than at 16.12 kPa (Figures 1I,K), whereas the expression of *Puma* was significantly lower at 14.13 kPa pO_2_ and that of *Apaf1* remained unaltered under different pO_2_ conditions (Figure 1J **, 1L**).

Taken together, the results demonstrate that apoptosis-related genes are differently regulated depending on the tissue and that, in some instances, have opposite expression patterns between plateau zokors and Sprague-Dawley rats.

### Homology Analysis of Apoptosis-Related Genes of Plateau Zokor

The complete coding sequence (CDS) of *Pidd* was 2,757 bp, which encoded a protein with 918 amino acid residues. Homology analysis showed that the CDSs of plateau zokor *Pidd* shared: 91.50%, 80.05%, 85.05%, 85.78%, 74.90%, 75.41%, and 79.78% nucleotide sequence homology (Table 1), and 94.56%, 79.85%, 85.87%, 86.60%, 74.40%, 74.84%, and 79.62% amino acid sequence homology (Table 2) with those of *N. galili*, *H. glaber*, *R. norvegicus*, *M. musculus*, *O. curzniae*, *O. princeps*, and *H. sapiens*, respectively.

**TABLE 1:** Sequence homology of apoptosis-related genes between plateau zokor and other species.

Genes	Length	*Nannospalax galili* (%)	*Heterocephalus glaber* (%)	*Rattus norvegicus* (%)	*Mus musculus* (%)	*Ochotona curzoniae* (%)	*Ochotona princeps* (%)	*Homo sapiens* (%)
*Pidd*	2,757	91.50	80.05	85.05	85.78	74.90	75.41	79.78
*Puma*	582	95.19	79.37	92.27	90.89	87.80	87.69	90.89
*Igfbp3*	879	93.64	68.97	87.95	87.39	54.20	55.09	82.57
*Apaf1*	3,750	94.00	88.39	87.54	88.23	78.22	80.38	88.98

**TABLE 2:** Amino acid sequence homology of apoptosis-related genes between plateau zokor and other species.

Genes	Length	*Nannospalax galili* (%)	*Heterocephalus glaber* (%)	*Rattus norvegicus* (%)	*Mus musculus* (%)	*Ochotona curzoniae* (%)	*Ochotona princeps* (%)	*Homo sapiens* (%)
*Pidd*	918	94.56	79.85	85.87	86.60	74.40	74.84	79.62
*Puma*	193	96.37	80.86	95.34	94.82	87.56	86.60	92.75
*Igfbp3*	292	96.23	69.28	84.59	85.96	55.97	55.63	80.94
*Apaf1*	1,249	94.00	88.39	90.71	91.51	77.10	79.02	88.15

The CDS of *Puma* was found to have a length of 582 bp, encoding a protein with 193 amino acid residues. These sequences shared: 95.19%, 79.37%, 92.27%, 90.89%, 87.80%, 87.69%, and 90.89% nucleotide sequence homology (Table 1), and 96.37%, 80.86%, 95.34%, 94.82%, 87.56%, 86.60%, and 92.75% amino acid sequence homology (Table 2) with those of *N. galili*, *H. glaber*, *R. norvegicus*, *M. musculus*, *O. curzniae*, *O. princeps*, and *H. sapiens*, respectively.

The CDS of *Igfbp3* was 879 bp, encoding a protein consisting of 292 amino acid residues. Homology analysis displayed that the CDS of plateau zokor *Igfbp3* shared: 93.64%, 68.97%, 87.95%, 87.39%, 54.20%, 55.09%, and 82.57% nucleotide sequence homology (Table 1), and 96.23%, 69.28%, 84.59%, 85.96%, 55.97%, 55.63%, and 80.94% amino acid sequence homology (Table 2) with those of *N. galili*, *H. glaber*, *R. norvegicus*, *M. musculus*, *O. curzniae*, *O. princeps*, and *H. sapiens*, respectively.

Lastly, the CDS of *Apaf1* was found to have a length of 3,750 bp, which encoded a protein of 1,249 amino acid. These sequences shared: 94.00%, 88.39%, 87.54%, 88.23%, 78.22%, 80.38%, and 88.98% nucleotide sequence homology (Table 1), and 94.00%, 88.39%, 90.71%, 91.51%, 77.10%, 79.02%, and 88.15% amino acid sequence homology (Table 2) with those of *N. galili*, *H. glaber*, *R. norvegicus*, *M. musculus*, *O. curzniae*, *O. princeps*, and *H. sapiens*, respectively.

### Positive Selection Site Analysis

To detect the positively selected sites of apoptosis-related genes in plateau zokor, we treated the plateau zokor branch as the foreground branch and used the branch-site model. The analysis of the apoptosis-related genes showed that two positive selection sites existed in Pidd of plateau zokor, that were Arg853 and Val898, respectively, and the LRT statistic comparing model A with the null model was not of statistical significance; one site in Puma of plateau zokor, there was Gln161, and the LRT statistic of model A with null model was not of statistical significance. Apaf1 and Igfbp3 had no positive selection sites in plateau zokor (*p* > 0.05) (Table 3).

**TABLE 3:** Likelihood ratio test (LRT) of branch-site models for apoptosis-related genes in plateau zokor.

Genes	Model	Estimate of parameters	-lnL^a^	Model comparison	Positively selected sites	2ΔlnL (*p* value)
Pidd	Null A	p0 = 0.76, p1 = 0.24, (p2+ p3 = 0.00), ω0 = 0.07, ω1 = 1.00, ω2 = 1.00	-19516.5			
	Model A	p0 = 0.76, p1 = 0.24, (p2+ p3 = 0.00), ω0 = 0.07, ω1 = 1.00, ω2 = 1.00	-19516.5	Model A vs. Null A	853 R, 898 V	0 (p = 1)
Puma	Null A	p0 = 0.93, p1 = 0.07, (p2+ p3 = 0.00), ω0 = 0.09, ω1 = 1.00, ω2 = 1.00	-2,809.95			
	Model A	p0 = 0.93, p1 = 0.07, (p2+ p3 = 0.00), ω0 = 0.09, ω1 = 1.00, ω2 = 1.00	-2,809.95	Model A vs. Null A	161 Q	0 (p = 1)
Igfbp3	Null A	p0 = 0.90, p1 = 0.10, (p2+ p3 = 0.00), ω0 = 0.09, ω1 = 1.00, ω2 = 1.00	-5,777.96			
	Model A	p0 = 0.90, p1 = 0.10, (p2+ p3 = 0.00), ω0 = 0.09, ω1 = 1.00, ω2 = 1.00	-5,777.96	Model A vs. Null A		0 (p = 1)
Apaf1	Null A	p0 = 0.86, p1 = 0.13, (p2+ p3 = 0.03), ω0 = 0.10, ω1 = 1.00, ω2 = 1.00	-21934.7			
	Model A	p0 = 0.86, p1 = 0.13, (p2+ p3 = 0.03), ω0 = 0.10, ω1 = 1.00, ω2 = 1.00	-21934.7	Model A vs. Null A		0 (p = 1)

### Convergent Evolution Analysis

**FIGURE 2:**
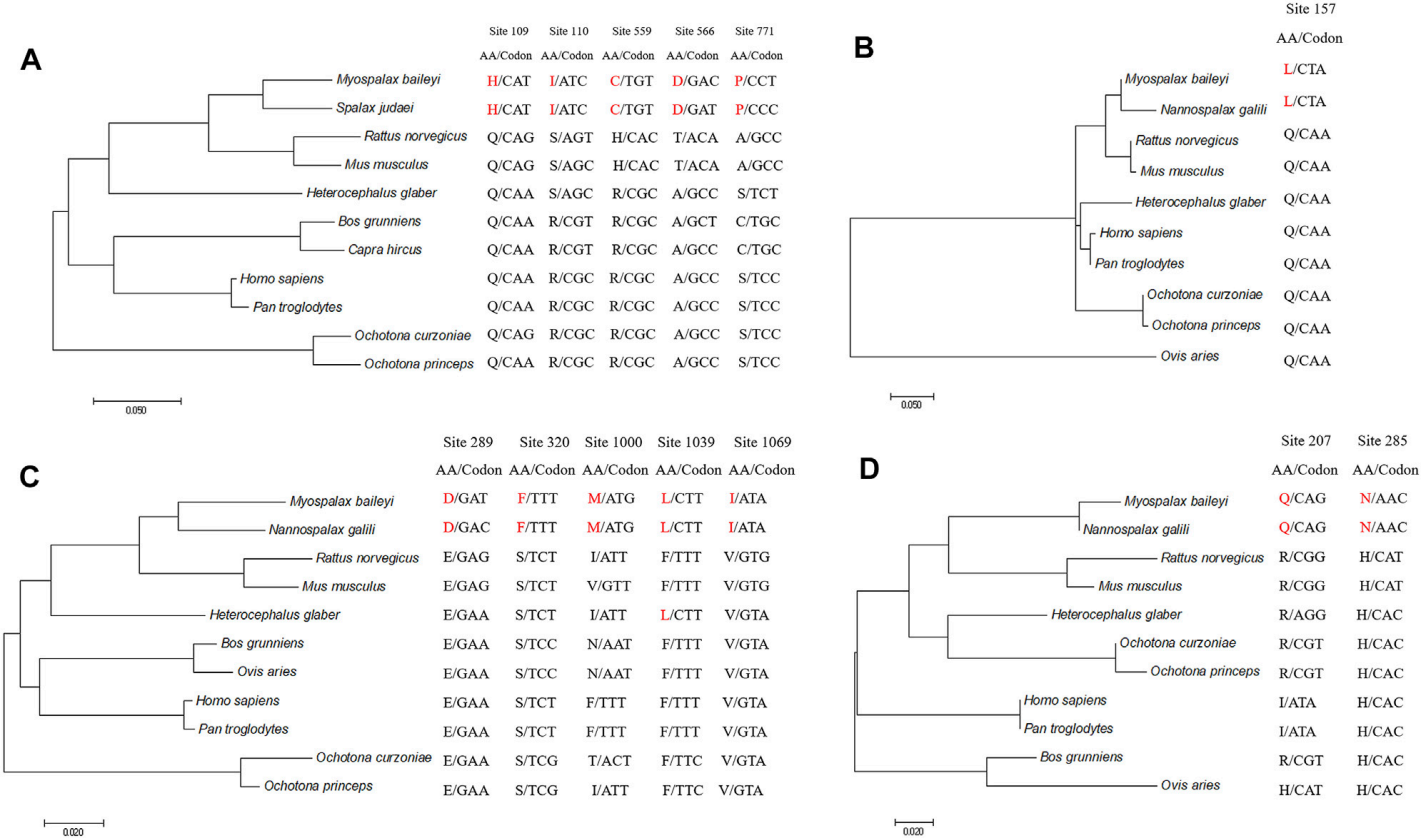
Evolution of convergent sites in Pidd, Puma, Apaf1, and Ifbp3 sequences. Panels indicate Pidd **(A)**, Puma **(B)**, Apaf1 **(C)** and Igfbp3 **(D)**. Amino acids and codons of convergent sites are shown. Amino acids in *Myospalax baileyi* and *Nannospalax galili* are highlighted in red.

To detect the convergent sites of apoptosis-related genes in subterranean rodents (*M. baileyi* and *N. galili*), we compared ancestral and extant amino acid sequences. Pidd was found to have five sites that underwent convergent evolution in subterranean rodents: the amino acids at positions 109, 110, 559, 566, and 771 were glutamine (Q), arginine (R), arginine (R), alanine (A), and serine (S) in the ancestral branch, respectively, which were replaced by histidine (H), isoleucine (I), cysteine (C), aspartic acid (D), and proline (P) in the subterranean rodent branch (Figure 2A). Puma had one site (position 157) that showed convergent evolution, as it was glutamine (Q) in the ancestral branch and it was replaced by lysine (L) in the subterranean rodent branch (Figure 2B). Moreover, Apaf1 had five sites that could experience convergent evolution in subterranean rodents: the amino acid positions 289, 320, 1,000, 1,039, and 1,069 were glutamic acid (E), serine (S), isoleucine (I), phenylalanine (F), and valine (V) in the ancestral branch, but were replaced by aspartic acid (D), phenylalanine (F), methionine (M), lysine (L), and isoleucine (I) in the subterranean rodent branch, respectively (Figure 2C). Lastly, Igfbp3 was found to have two sites of possible convergent evolution (positions 207 and 285) in which an arginine (R) and histidine (H) in the ancestral branch were replaced by a glutamine (Q) and asparagine (N) in the subterranean rodent branch, respectively (Figure 2D).

According to a statistical test, these identified convergent sites were confirmed to be likely convergent sites, rather than chance substitutions (*p* < 0.01).

### Protein Structure Homology Modeling

**FIGURE 3:**
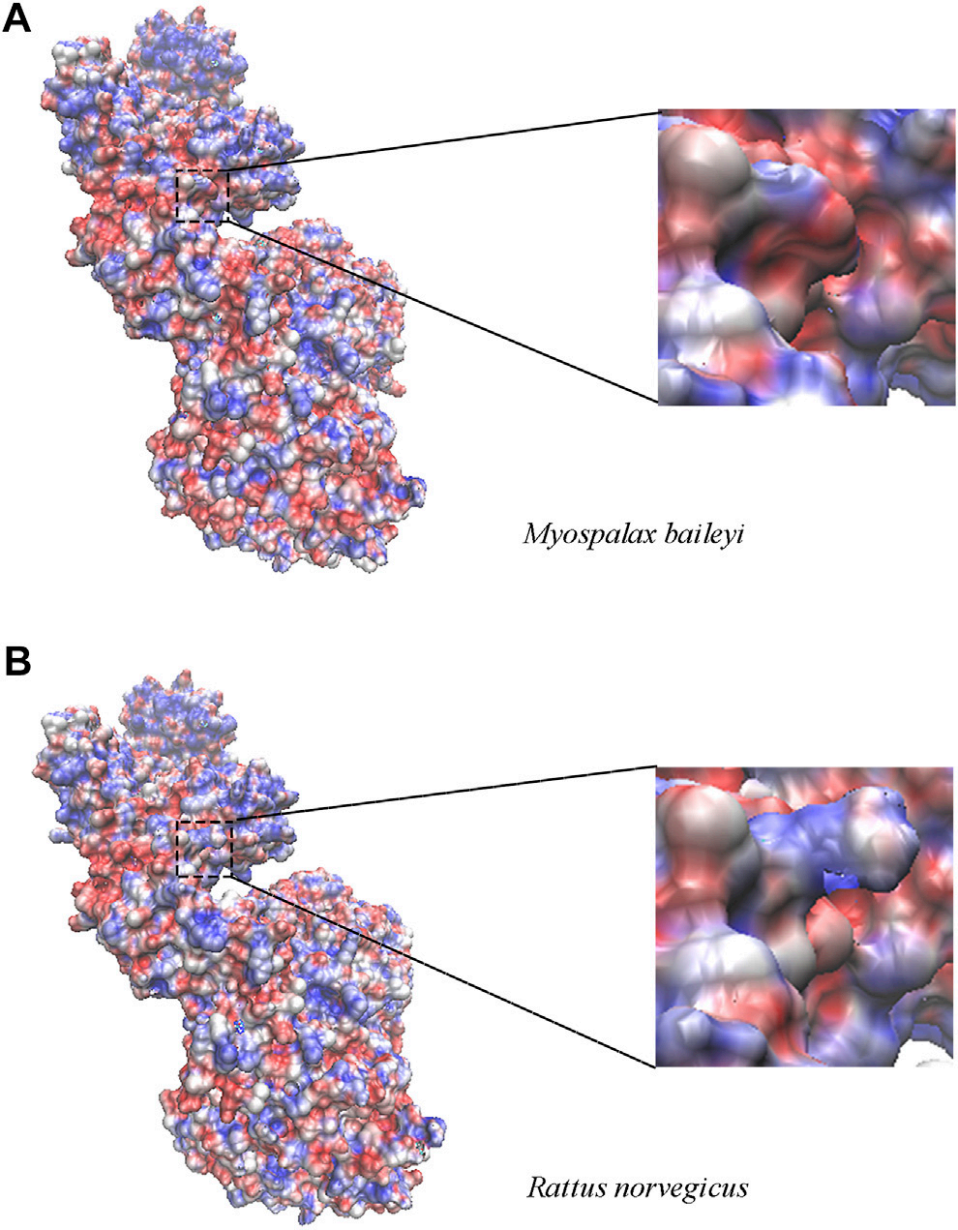
The electrostatic potential map of Apaf1 in plateau zokor (*Myospalax baileyi*) **(A)** and Sprague–Dawley rat (*Rattus norvegicus*) **(B)**. The range of surface electrostatic potential was shown from -7 kT/e to 7 kT/e in red and blue, respectively. At the right panel, the surface electrostatic potential around the substitution sites was enlarged at residue 320.

Next, we investigated the relationship between the amino acid convergent sites and the structure changes with functional properties in the apoptosis-related genes. Unfortunately, only the high homology model of *Apaf1* was available at the SWISS-MODEL server; thus, we were unable to do this analysis for *Pidd*, *Puma*, and *Igfbp3*. The convergent site at position 320 of Apaf1 was a serine (S) in *R. norvegicus*, whereas was substituted by phenylalanine (F) in plateau zokors, and was located in the central CED-4 domain. Since the polar amino acid (S) was substituted by a non-polar amino acid (F), the regional electrostatic potential changed in enlarged electrostatic potential maps (Figure 3).

## Discussion

The plateau zokor has a strong adapting capability to the hypoxic environment of its burrows. In the present study, we measured the levels of apoptosis-related genes in plateau zokor and Sprague-Dawley rats at different oxygen partial pressures. Overall, we found that the *Pidd* levels were downregulated in the lung of plateau zokor when under low pO_2_. In contrast, low pO_2_ upregulated *Pidd* expression in the liver and skeletal muscle of Sprague-Dawley rats. *Puma*, *Igfbp3*, and *Apaf1* are proapoptotic genes involved in the mitochondria-dependent apoptotic pathway. Noteworthy, low pO_2_ downregulated the expression of *Puma* in the lung, whereas was upregulated in the skeletal muscle of plateau zokor. Moreover, low pO_2_ also upregulated the levels of *Igfbp3* and *Apaf1* in the tissues of plateau zokor. In turn, in Sprague-Dawley rats, low pO_2_ downregulated the levels of *Igfbp3* and *Apaf1* in the lung and liver, respectively, and upregulated the levels of *Igfbp3* in the skeletal muscle. Taken together, plateau zokors and Sprague-Dawley rats showed opposite expression patterns of *Apaf1*, *Igfbp3*, and *Puma* in the liver, lung, and skeletal muscle, respectively. A previous study illustrated that the transactivation of *Puma* is downregulated in human non-small cell lung carcinoma cells transfected with wild-type p53 or N104S mutated p53 of plateau zokor (Zhao et al., 2013). In particular, p53^N104S^ harboring cells displayed upregulated *Igfbp3* transactivation under hypoxic stress (0.2% O_2_) (Zhao et al., 2013). In the subterranean rodent *Lasiopodomys mandarinus*, *Igfbp3* was reported to be upregulated under hypoxic conditions (Li, 2017). Another study in *Spalax* also showed that the mRNA levels were decreased under hypoxic conditions upon impaired transcription of *Apaf1*, whereas they were increased in the rat (Band et al., 2010). In addition, p53 upregulates the transactivation of plateau zokor *Apaf1* under hypoxic conditions in *in vitro* experiments (Zhao et al., 2013). Therefore, low oxygen partial pressures induce apoptosis by upregulating the apoptosis-related genes in tissues of plateau zokor, so as to prevent tumorigenesis under hypoxic environments. Moreover, the apoptosis-related genes demonstrated tissue-specific, differential expression patterns in plateau zokor, which reflects the differentiation of the tissues and their physiological correlation with a hypoxic adaptation of metabolic, respiratory, and energy regulation. Furthermore, we observed different expression patterns of the apoptosis-related genes between plateau zokor and Sprague-Dawley rats. Studies have reported that subterranean rodents harbor amino acid substitutions in hemoglobin, myoglobin, and cytoglobin, which resulted in increased affinity toward oxygen and an enhanced capacity to adapt to hypoxic environments (Gurnett et al., 1984; Kleinschmidt et al., 1984). Thus, we hypothesize that the different expression patterns noted in plateau zokor and Sprague-Dawley rats are closely related to the sequence homology and substitution sites.

Herein, homology analysis showed that the nucleotide and amino acid sequences of the apoptosis-related genes in plateau zokor were high similar to those of *N. galili*, followed by *R. norvegicus*. This observation may be explained by the fact that *N. galili* and plateau zokor belong to the family of subterranean rodents; thus, they may have developed similar strategies to adapt to the hypoxic-hypercapnic burrowing environments (Arieli and Ar, 1981; Zeng et al., 1984; Arieli et al., 1986; Widmer et al., 1997; Shao et al., 2015). Indeed, phylogenetic studies based on the transcriptome and genome of plateau zokor showed that they diverged from the rat approximately 52 million years ago (Shao et al., 2015).

Pidd is a molecular switch between cell survival and apoptosis (Wu et al., 2005), which comprises seven leucine-rich repeats (LRRs) at the N-terminus, followed by two ZO-1 and Unc5-like (ZU-5) domains and a C-terminal death domain (DD) (Tinel and Tschopp, 2004). The full-length Pidd protein is constitutively cleaved into three fragments: Pidd-N (1–445aa, 48 kDa), which contains the N-terminal LRRs and the proximal ZU-5; Pidd-C (446–910aa, 51 kDa), which contains the distal ZU-5 and DD; and Pidd-CC (588–910aa, 37 kDa), which contains the DD (Tinel and Tschopp, 2004; Tinel et al., 2007). Evolution analysis displayed that the Pidd of plateau zokor and *N. galili* had five convergent sits (H109, I110, C559, D566, and P771). H109 and I110 were located in the LRRs at the N-terminus, C559 and D566 were located in the DD, and P771 was in the Pidd-CC fragment. Previous studies have shown that Pidd-N may be involved in a stress-related signal. Moreover, Pidd-CC interacts with receptor-interacting protein (RIP)-associated ICH-1/CED-3 homologous protein with a DD, as well as with caspace-2 to form a protein complex, named PIDDosome, which will, in turn, trigger the activation of mitochondria-dependent apoptosis (Tinel and Tschopp, 2004; Berube et al., 2005; Ren et al., 2005). However, Pidd-C mediates the activation of NF‐κB via recruitment of RIP1-1 and NF-κB essential modulator (NEMO), resulting in apoptosis inhibition (Janssens et al., 2005). Since a positively charged histidine (H) substituted the cysteine (C) at position 559, the threonine (T) substituted for negatively charged aspartic acid (D) at the position 566, the regional electrostatic potential was, thus, the substitution may have altered Pidd-C affinity for RIP1 and NEMO, as well as and the activity of NF‐κB, resulting in decreased ability to inhibit apoptosis in plateau zokor tissues. Puma is a proapoptotic BH3-only member of the Bcl-2 protein family that induces apoptosis via its BH3 domain but also by the mitochondrial targeting sequences (MTS) located in its C-terminus, the deletion of MTS could not be recognized the mitochondrial so that apoptotic induction was lost (Nakano and Vousden, 2001; Yu et al., 2001; Steckley et al., 2007). In the present study, evolution analysis showed that plateau zokor Puma had one convergent evolution site at position 157, with the substitution of a polar amino acid (Q) by a non-polar amino acid (L) in its MTS domain compared with the subterranean rodent of *N. galili*. We hypothesized that the upregulated levels of *Puma* in the skeletal muscle of plateau zokor, and the different expression pattern of *Puma* in skeletal muscle was closely related to the convergent evolution site.

*Igfbp3* is a hypoxia-inducible gene, which transcription is activated through the hypoxia-inducible factor-1α (Natsuizaka et al., 2012). In this study, we observed that two convergent evolution sites occurred in Igfbp3 of plateau zokor and *N. galili* at the residues Q207 and N285 in the C-terminus. However, compared with these two subterranean rodents, the amino acids in the same sites in the sequence of Sprague-Dawley rat Igfbp3 were arginine (R) and histidine (H). Igfbp3 regulates apoptosis in an insulin-like growth factor (IGF)-dependent manner, through the IGF-binding sites at the N- and C-terminal regions (Spencer and Chan, 1995; Ho and Baxter, 1997; Devi et al., 2000; Imai et al., 2000; Buckway et al., 2001). Studies have shown that the affinity of Igfbp3 for IGF-I/II was lost when its residues I56, L80, and L81 were substituted by glycine (G), which prevented apoptosis and also promoted the tumor growth *in vivo* and *in vitro* (Buckway et al., 2001; Takaoka et al., 2007; Natsuizaka et al., 2012). Low pO_2_ showed to upregulate *Igfbp3* levels in the tissues of plateau zokor, whereas it downregulated *Igfbp3* in the lung of Sprague-Dawley rats, indicating that the substitution of the two convergent sites may change the affinity of the IGF and regulate *Igfbp3* transcription under low oxygen concentration. Apaf1, along with cytochrome c and dATP, forms the apoptosome complex that then recruits caspase 9 (via its caspase recruitment domain) to induce apoptosis (Bratton and Salvesen, 2010; Yuan and Akey, 2013). Our results displayed that Apaf1 has five convergent evolution sites (D289, F320, M1,000, L1,039, and I1,069) in plateau zokor. The M1,000, L1,039, and I1,069 sites were in the C-terminal regulatory Y-domain, which is composed of 12–13 WD40 repeats (WDRs), and D289 and F320 were located in the central CED-4 domain. Previous studies have demonstrated that mutations in the WDRs and CED-4 affect the ability of Apaf1 to bind to cytochrome c and caspase9, and induce apoptosis (Srinivasula et al., 1998). Mutagenesis studies also showed that the Apaf1 variant lacking the WDRs was still able to activate procaspase-9 independently of cytochrome c and dATP (Hu et al., 1999). Moreover, the M368L (in the CED-4 domain) mutated variant is more potent inducing apoptosis than the wild-type Apaf1 (Hu et al., 1998; Hu et al., 1999). Herein, the S320F was found to lead to regional electrostatic changes in the electrostatic potential map. In addition, Low pO_2_ upregulated *Apaf1* expression in the liver and lung of plateau zokor. *Apaf1* expression pattern in liver differed between plateau zokor and Sprague-Dawley rats. Taken together, these findings suggest that *Apaf1* expression is closely related to the convergent evolution sites. Nevertheless, the function of the convergent evolution sites of apoptosis-related genes warrants further investigations by site-directed mutagenesis technology.

## Conclusion

For the long-time adaptation to the hypoxic environments, apoptosis-related genes of plateau zokor underwent convergent evolution, with convergent evolution sites potentially playing an important role in controlling gene expression. Indeed, the convergent evolution sites of the apoptosis-related genes may be responsible for the upregulation of their levels under low oxygen partial pressures to induce apoptosis in tissues of plateau zokor and consequently suppress tumorigenesis under hypoxic environments.

## Data Availability

The original contributions presented in the study are included in the article/Supplementary Materials; further inquiries can be directed to the corresponding author.
